# Effects of Chemical Additives on Viable Bacterial Count and Physicochemical Parameters of Water Used during Live Transportation of Climbing Perch (*Anabas testudineus*)

**DOI:** 10.1002/vms3.70544

**Published:** 2025-08-11

**Authors:** Maliha Afsana, Md. Nazmul Islam Rifat, Md. Mubrack Hossain, Md. Naim Uddin, Md. Nurul Haider

**Affiliations:** ^1^ Department of Fisheries Technology Bangladesh Agricultural University Mymensingh Bangladesh

**Keywords:** *Anabas testudineus*, bacterial regrowth, Climbing perch, live transportation, water additives, water quality parameters

## Abstract

**Background:**

During live transportation of Climbing perch (*Anabas testudineus*), stressors and bacterial regrowth pose significant challenges for animal welfare and lead to decline in fish quality. Some chemical additives are commonly used in transport water; however, their efficacy and doses are not well established.

**Objectives:**

This study aimed to evaluate the efficiency of different water additives used during live transportation of Climbing perch, in terms of their effects on water quality, bacterial regrowth and degree of stress imposed on fish.

**Methods:**

Experiments were conducted under two groups: (1) on‐farm under laboratory conditions and (2) on‐field in a real fish supply channel from the production sites to the retail market. In the on‐farm experiment, tank waters were treated with some additives such as salt, methylene blue (MB) and electromin saline (ES), separately and in combination. The most effective additives and their dosages obtained after on‐farm experiment were applied in the on‐field experiment. Changes in water quality parameters, bacterial load and the degree of stress imposed on fish were followed.

**Results:**

Treated tanks showed improved outcomes in terms of maintaining better water quality, significantly reducing bacterial regrowth and alleviating fish stress. Concentrations of 2.0 ppt salt, 2.0 ppt saline, 1.0 ppm MB and a combination of 2.0 ppt saline + 1.0 ppm MB proved to be highly effective in combating poor water quality, stressful conditions and bacterial regrowth during live transport of Climbing perch.

**Conclusions:**

The combination of MB with ES/salt was found effective and could be adopted by fish farmers and traders to minimize fish quality deterioration, mortality and financial losses.

## Graphical abstract

The efficiencies of different water additives, in terms of their impacts on water quality, bacterial regrowth and stress on fish, were evaluated during live transportation of Climbing perch (*Anabas testudineus*). Additives like salt, methylene blue and electromin saline were found effective and could be adopted by the fish farmers/traders.

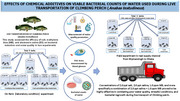

## 1 Introduction

Live fish transportation is essential worldwide, particularly for human consumption (Marcalo et al. 2013). Live fish generate a higher market price because of their greater demand from consumers (Harmon 2009). Thus, transportation of live fish to different regions or retail markets is becoming a common practice among the fish producers and traders. Climbing perch, *Anabas testudineus*, is one of the most important aquaculture species in Bangladesh (Chhanda et al. 2019), which can be cultured in both freshwaters and brackish waters (Pethiyagoda et al. 2008). An increasing number of farmers across the country produce Climbing perch both under polyculture and monoculture systems (Craig et al. 2004), and in recent years, live transportation of Climbing perch is becoming popular (Hossain et al. 2021, 2024). Although Climbing perch is a relatively harder fish and has accessory respiratory organ, their physical injury and skin lesion are common consequence during live transportation, which ultimately results in reduced market price and demand. Giving proper attention during live transportation of Climbing perch is necessary to avoid any sort of quality deterioration and keep their market demand.

In many cases, fish survival during live transportation to the destination is challenged because of higher density, continuous changes in water quality and bacterial regrowth (Lekang 2007; Espinosa‐Curiel et al. 2016; Hossain et al. 2021). The duration of transportation can vary, typically being either short (2 h) (Sherif et al. 2023) or long (up to 72 h) (Zhang et al. 2023), which is frequent in Bangladesh. Commonly fishes are transported via trucks or cars worldwide (Azambuja et al. 2011; Correia et al. 2011; Bortoletti et al. 2023). As a common practice in Bangladesh, plastic barrels filled with underground water are loaded to commercial trucks for live transportation of fishes from the production sites to the retail markets (Hossain et al. 2021; Bhuiyan et al. 2022).

During the transport, several factors have the potential to create physiological changes that affect fish health (Harmon 2009). The physicochemical parameters of water such as temperature, dissolved oxygen (DO), ammonia and pH are the most common factors that act as stressors and affect the physiological condition of the live fishes (Golombieski et al. 2003; Stefansson et al. 2007; Carneiro et al. 2009). Moreover, bacteria introduced into the transport water through the pond water and fish itself regrow and increase in numbers depending on the distance from the loading points to the final destination (Hossain et al. 2021). Poor water qualities reduce body slime, damage scales and impose stress to the fish and finally make them more susceptible to the diseases (Francis et al. 2002). Longer transport event also increases the concentration of the suspended solids and results temperature fluctuation (Harmon 2009; Crosby et al. 2005), which may increase the degree of stress to the fishes.

Again, increases of O_2_ consumption and associated developments in CO_2_ excretion result in transport‐induced stress to the fishes during live transportation (Iversen et al. 1998). Fish exhibit a series of physiological stress responses, including release of cortisol into the bloodstream as a primary reaction, then elevated blood glucose levels (often used as a parameter to measure degree of stress), and finally, physiological adjustments as long‐term tertiary responses, if the stress continues (Barton 2002; Gomes et al. 2003; Acerete et al. 2004; Iwama et al. 2006; Urbinati and Carneiro 2006; Sampaio and Freire 2016). When fish remain in a high level of stress, they become more susceptible to bacterial attacks (Dobšíková et al. 2006), which can result in a higher degree of quality deterioration and even mortality (Barton et al. 2005).

Thus, maintaining proper water quality during live fish transportation is a vital consideration in reducing physiological stress and diminishing fish mortality (Harmon 2009; Refaey et al. 2018). In order to keep and/or enhance the water quality during live fish transportation, several water additives, such as sodium chloride/table salt (NaCl), commercially available salines and methylene blue (MB) (C_16_H_18_N_3_CIS.3H_2_O), are commonly used (Fajardo 2002; Crosby et al. 2005; Chuan et al. 2007; Harmon 2009; Oz et al. 2011). Salt is inexpensive, readily available and, when properly administered, safe for use in freshwater fish. Salt (NaCl) is a stress mitigator of fish transportation (Tacchi et al. 2015) and decreases the maximum physiological responses to stress showing no mortality (Carneiro and Urbinati 2002). Salt can also lower the mortality rate of transported live fish by maintaining water quality (Hansen 2009). It was reported that MB is inhibitory to microorganisms (Oz et al. 2011). Combined use of MB and salt showed even better results against bacteria during live transportation fish (Bolivar et al. 2004). Commercially available saline and MB are bacterial inhibitors and recommended to use during live fish transportation (Faruk et al. 2019; Yeasmin et al. 2015). These chemical additives are commonly used during live transportation of Climbing perch in Bangladesh to avoid stress, enhance water quality parameters and avoid bacterial regrowth. However, the efficiency of these chemical compounds as well as their appropriate doses is not well established.

The study aimed to evaluate the impact of various water additives, including salt, commercially available electromin saline (ES) and MB, on bacterial regrowth/viable counts and water quality parameters and the degree of stress on fish. Additionally, it sought to determine the most effective dosages of these additives for transporting live Climbing perch over different time intervals.

## 2 Materials and Methods

### 2.1 Experimental Sites and Laboratory Used

**FIGURE 1:**
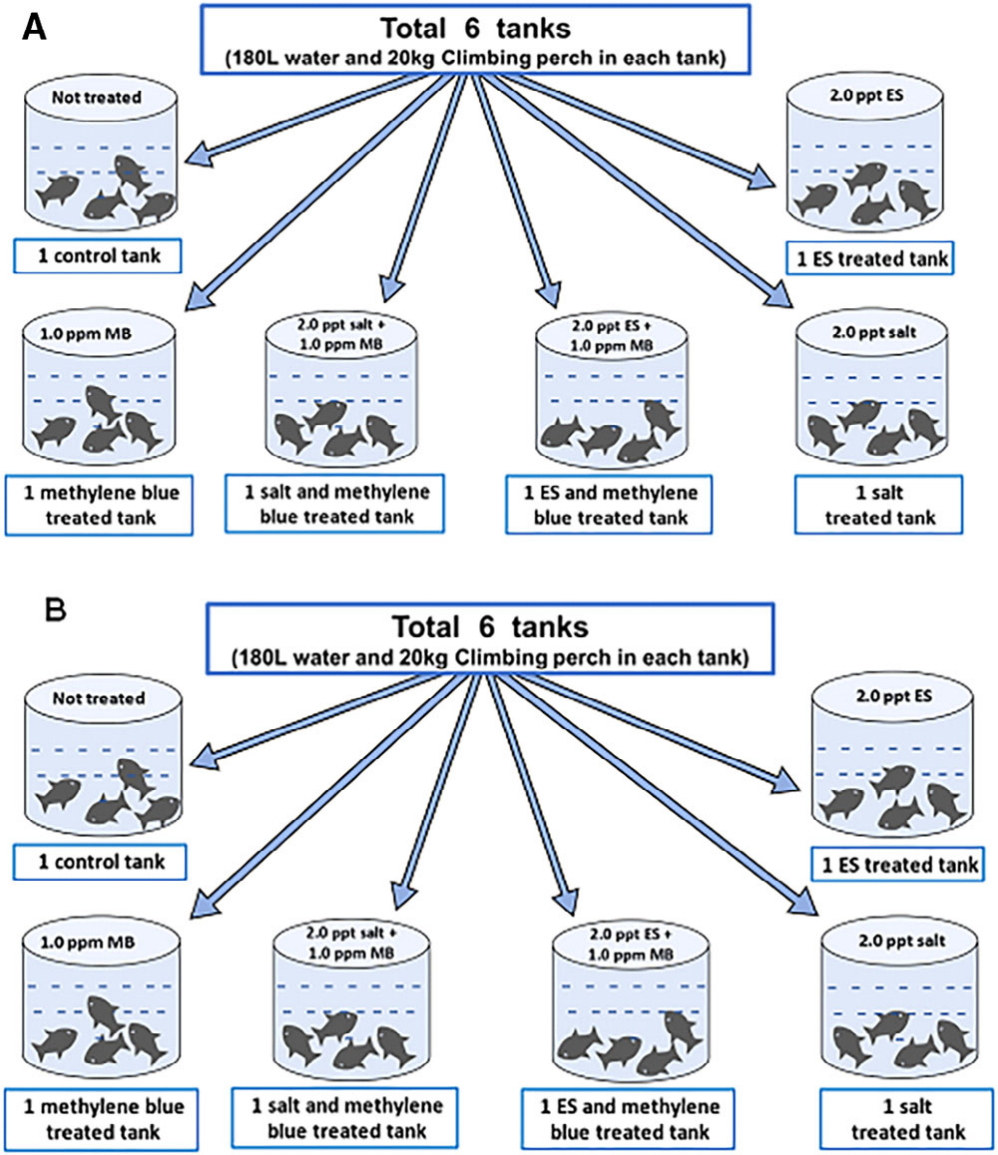
Experimental design used to evaluate the effects of chemical additives on viable bacterial count and physicochemical parameters of water used during live transportation of climbing perch (*Anabas testudineus*): (A) on‐farm (laboratory condition) experiment and (B) in field experiment. ES, electromin saline; MB, methylene blue.

Two different sets of experiment were conducted on‐farm in laboratory condition (Figure 1A) and on field in a real supply channel (Figure 1B) from April 2022 to July 2022. Initially, the experiment of laboratory condition was set up under a shade on‐farm (Figure 1A). For the field experiment, another experiment was conducted in a real supply channel from the production site to a retail market (Trishal Upazila under Mymensingh district to Karwan Bazar, Dhaka) (Figure 1B). Further activities were performed in the Fisheries Microbiology Laboratory of Department of Fisheries Technology, Faculty of Fisheries, Bangladesh Agricultural University, Mymensingh.

### 2.2 Harvesting and Experimental Setup

Climbing perch were harvested from a pond by using surrounding net. The harvested fishes were then kept within a net barrier in one corner of the pond and then transferred to the plastic tanks for treatment.

For on‐farm (laboratory condition) experiment, nine plastic tanks were placed under a shade. Each tank contained 18 L of deep tube‐well water and approximately 2 kg (8–10 fishes per kg) of live Climbing perch to make a mimic situation as practicing in the supply channels for retailing. The fishes were acclimatized before delivering to the experimental tanks. Then, the tanks were treated with table salt (2.0 ppt); commercially available ES (electromin powder, Square Pharmaceuticals Ltd., Bangladesh; contains sodium bicarbonate, sodium chloride, potassium chloride, dextrose, vitamin A, zinc sulphate, calcium chloride and manganese sulphate) (2.0 ppt); MB (0.5, 1.0, and 1.5 ppm) and combination of salt and MB (2.0 ppt salt + 0.5 ppm MB, 2.0 ppt salt + 1.0 ppm MB and 2.0 ppt salt + 1.5 ppm MB) (Faruk et al. 2019). One tank was kept as control without any treatment. To assess the viable bacterial count, water samples were collected at 2 h intervals up to 8 h.

For the field experiment, six plastic tanks were treated with the best doses of the chemicals found from the laboratory experiment. The tanks were placed on a truck containing around 20 kg of live Climbing perch and 180 L of deep tube‐well water. The tanks were treated with 2.0 ppt table salt, 2.0 ppt commercially available ES, 1.0 ppm MB, combination of 2.0 ppt salt + 1.0 ppm MB and combination of 2.0 ppt ES + 1.0 ppm MB (Faruk et al. 2019). One tank was kept as control without treatment. The doses of the treatments were determined on the basis of the previous reports and common practices by the transporters. The tanks were transported from Trishal, Mymensingh, to Karwan Bazar, Dhaka. To assess the viable bacterial count, water samples were collected at 2 h intervals.

### 2.3 Sample Collection

Water samples were taken from the surface layer of the fish holding tanks by using 250 mL sterilized plastic bottles. Samplings were done at 2 h intervals from loading the fish (0 h) for a period of 8 h in case of on‐farm experiment. Similarly in case of the field experiment (in supply channel), water samples were collected at 2 h intervals and continued until unloading the fish at the retail market.

### 2.4 Determination of Physicochemical Parameters

The physicochemical parameters of the transport water such as water temperature, DO, pH and NH_3_ were measured according to the manufacturers’ protocols as described by Hossain et al. (2021). Temperature of the transport water was determined by a mercury glass thermometer. DO content of the water was determined using a DO test kit (Biosol, AA Biotech, India) following the manufacturer procedure. The pH values of the water were determined by using pH test kit (CP‐102, Thailand) following the manufacturer protocol. However, ammonia content was determined using total ammonia test kit (AQUA AM) according to the protocol suggested.

### 2.5 Estimation of Viable Bacterial Counts

The collected water samples were serially diluted and placed onto agar plates (HIMEDIA, India). Before plating, the samples were diluted using 0.85% w/v NaCl. Then, 10 µL (0.01 mL) of each tenfold diluted water sample was transferred and dropped (here drop plate method was used) onto agar plates using a micropipette maintaining two replications. The cultured agar plates were then transferred to laboratory and incubated at 30°C for 24 h.

After incubation, plates of having 3–30 colonies on each segment of the agar plates were counted. The average number of colonies for a particular dilution was multiplied with dilution factor to obtain the viable bacterial counts. The result of the viable count of bacteria was expressed as the number of colony forming unit (CFU)/mL of water sample. The calculation formula is as follows:

Viable count of bacteria in water (CFU/mL) = no. of colonies on agar plate × dilution factor × 100.

### 2.6 Determination of Blood Glucose Levels of Live Fish

The blood glucose levels of the experimental fishes were determined by a blood glucometer (G‐425‐3 Easy, Bioland Technology Ltd., China). The fishes were gently handled and placed on a soft pad to collect the blood samples using a syringe and were immediately loaded to the glucometer. The blood glucose levels were monitored just before the loading of the fishes and at the end of the experimental period (approximately after 8 h of observation) for the both laboratory experiment (on‐farm) and field experiment (in a supply channel).

### 2.7 Statistical Analyses

Collected data were recorded and preliminarily analysed using MS Excel. To assess the effects of different chemicals/treatments, one‐way ANOVA was conducted using SPSS (version 25). The outputs were presented in tabular and graphical formats.

## 3 Results

### 3.1 Changes in Physicochemical Parameters of Live Climbing Perch Transport Water

**FIGURE 2:**
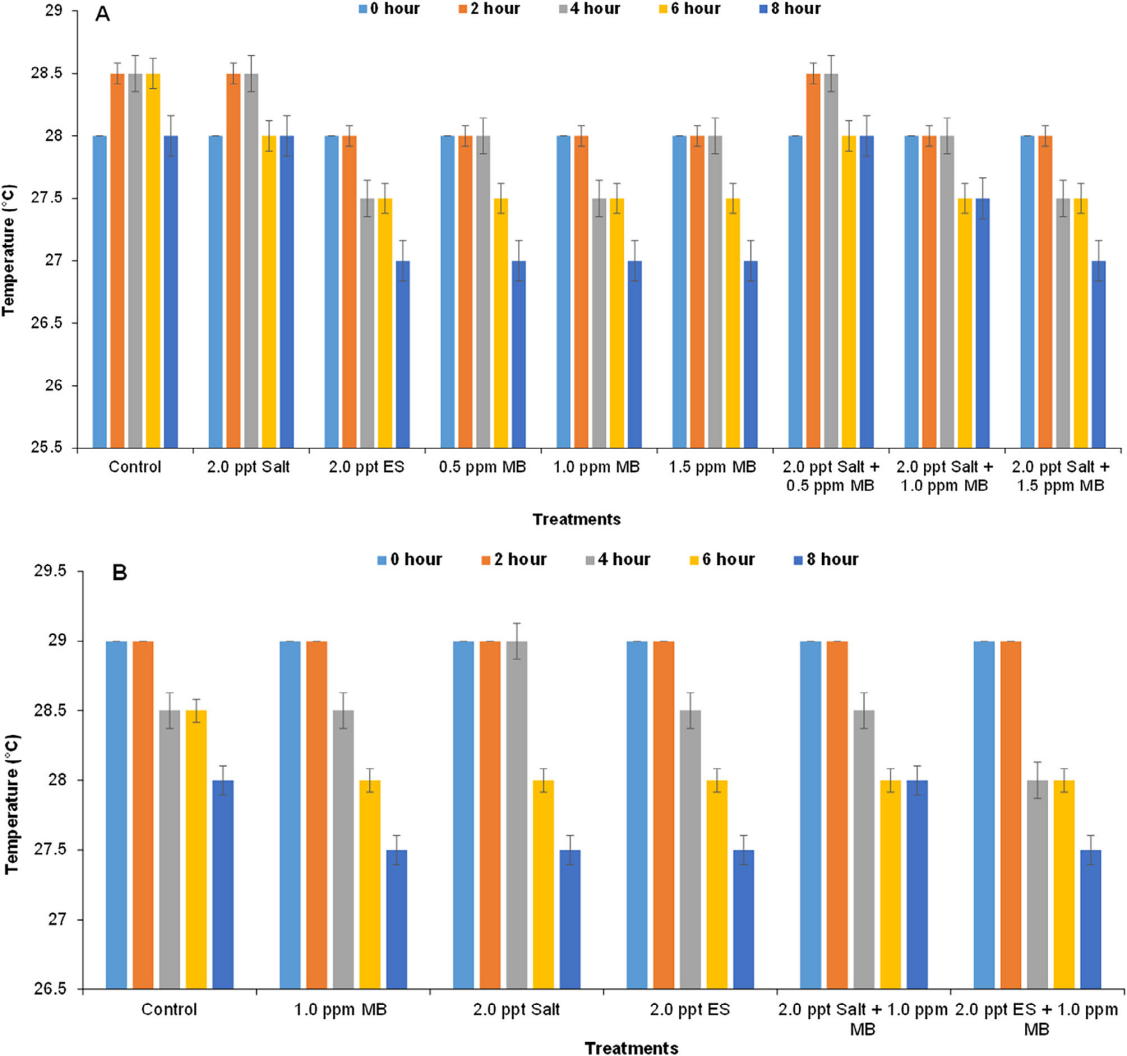
Fluctuations in temperatures of the water within the experimental tanks at different time intervals: (A) in case of on‐farm experiment and (B) in field experiment during live transportation of climbing perch (*Anabas testudineus*) treated with different water additives. ES, electromin saline; MB, methylene blue.

We found the temperature more or less similar in both laboratory experiment and field experiment. Basically, water temperature is fully dependent on atmospheric temperature and was fluctuated slightly (28.5–27.5°C) (Figure 2A). In the field experiment, the temperature was found between 29°C and 27.5°C in different treatments indicating no significant fluctuations (Figure 2B).

**FIGURE 3:**
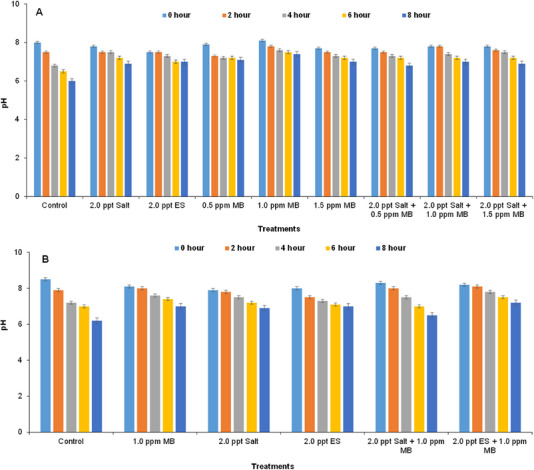
Changes in pH values of water in the experimental tanks at different time intervals: (A) in case of on‐farm experiment and (B) in field experiment during live transportation of climbing perch (*Anabas testudineus*) treated with different water additives. ES, electromin saline; MB, methylene blue.

Initial pH values were higher in all the treatments (∼8.0) and found to decrease slightly with time for both the laboratory (Figure 3A) and field (Figure 3B) experiments. However, the values became slightly lower in the control tanks after 8 h; values dropped from 8.0 to 6.0 (Figure 3A) in case of on‐farm experiment and from 8.5 to 6.2 (Figure 3B) in case of field experiment, respectively.

**FIGURE 4:**
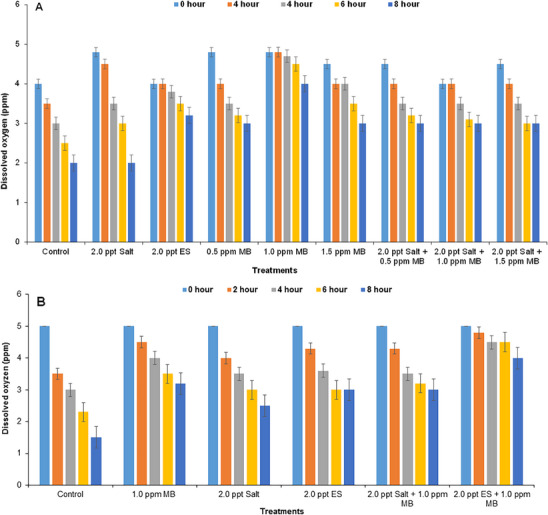
Changes of dissolved oxygen (DO) concentration of water in the experimental tanks at different time intervals: (A) in case of on‐farm experiment and (B) in field experiment during live transportation of climbing perch (*Anabas testudineus*) treated with different water additives. ES, electromin saline; MB, methylene blue.

Initially DO values were relatively higher and also similar in all treatments. DO values were decreased with time interval and become lowest after 8 h in both the cases of on‐farm (Figure 4A) and field experiment (Figure 4B). The DO level was lowest in the control tanks after 8 h, and the value ranged from 4.8 to 2 ppm (Figure 4A) and from 5 to 1.5 ppm (Figure 4B) in on‐farm and field experiment, respectively.

**FIGURE 5:**
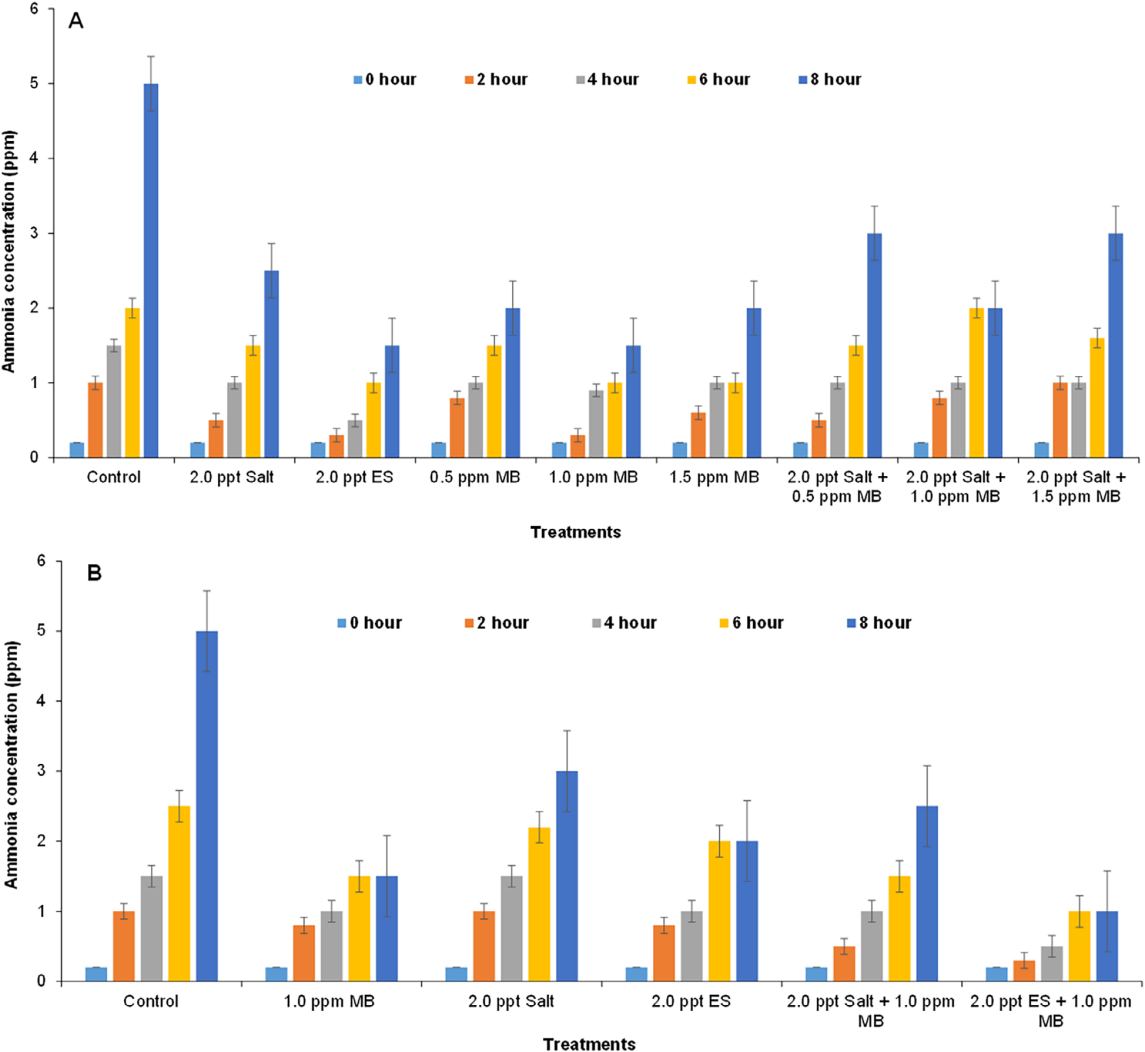
Fluctuations in ammonia concentrations of water in the experimental tanks at different time intervals: (A) in case of on‐farm experiment and (B) in field experiment during live transportation of climbing perch (*Anabas testudineus*) treated with different water additives. ES, electromin saline; MB, methylene blue.

The ammonia concentrations were relatively lower and similar in all treatments initially (Figure 5A,B). The concentrations were then increased with time interval especially after 8 h in both on‐farm (Figure 5A) and field experiments (Figure 5B) but did not fluctuate significantly. Relatively higher values were found in the control tanks compared to others especially after 8 h in both the cases and the values mostly fluctuated also in the control tanks in both on‐farm (Figure 5A) and field experiment (Figure 5B) and ranged from 0.2 to 5.0 ppm (Figure 5A,B).

### 3.2 Effects of Different Treatments on the Viable Bacterial Counts

In case of on‐farm experiment, bacterial viable counts were gradually increased (*p = *0.002) in the untreated tank/control tank, whereas in the treated tanks, the counts were found to decrease gradually (Table 1). In the control tank, initial viable counts were 2.25 ± 0.208 × 10^4^ CFU/mL, which become 3.03 ± 0.208 × 10^4^ CFU/mL at the end of the experiment (at 8 h). On the other hand, the bacterial counts in the MB‐treated water were decreased from 2.38 ± 0.298 × 10^4^ to 1.25 ± 0.298 × 10^4^ CFU/mL in 0.5 ppm MB, 2.23 ± 0.206 × 10^4^ to 1.05 ± 0.206 × 10^4^ CFU/mL in 1.0 ppm MB and 2.35 ± 0.238 × 10^4^ to 1.10 ± 0.238 × 10^4^ CFU/mL in 1.5 ppm MB‐treated tanks. Bacterial counts in other treatments were also found to decrease significantly from 2.43 ± 0.221 × 10^4^ to 1.73 ± 0.221 × 10^4^ CFU/mL in 2.0 ppt salt‐treated tank (*p *= 0.004) and from 2.28 ± 0.275 × 10^4^ to 1.35 ± 0.275 × 10^4^ CFU/mL in 2.0 ppt ES‐treated tank (*p *= 0.000). Combined use of salt and MB‐treated tanks was also found effective in lowering the bacterial viable counts, which were 2.10 ± 0.365 × 10^4^, 1.70 ± 0.365 × 10^4^ and 2.18 ± 0.238 × 10^4^ CFU/mL in the 2.0 ppt salt + 0.5 ppm MB, 2.0 ppt salt + 1.0 ppm MB and 2.0 ppt salt + 1.5 ppm MB‐treated tanks, respectively, after 8 h of observation (Table 1). At the end of the experimental periods, relatively lower counts were found in the tanks treated with 2.0 ppt salt, 2.0 ppt ES and 1.0 ppm MB, indicating that these doses of treatments reduced bacterial counts (Table 1).

**TABLE 1:** Changes in viable bacterial counts (CFU/mL) of water in case of on‐farm experiment.

	Viable count of bacteria (mean ± SD) × 10^4^ CFU/mL								
		Salt treated	Saline treated	Methylene blue treated			Combination of salt and methylene blue treated		
Experimental periods (h)	Control	2.0 ppt salt	2.0 ppt ES	0.5 ppm MB	1.0 ppm MB	1.5 ppm MB	2.0 ppt salt + 0.5 ppm MB	2.0 ppt salt + 1.0 ppm MB	2.0 ppt salt + 1.5 ppm MB
0	2.25 ± 0.20^a^	2.425 ± 0.20^b^	2.275 ± 0.27^b^	2.375 ± 0.29^c^	2.225 ± 0.22^c^	2.35 ± 0.23^c^	2.4 ± 0.36^a^	2.475 ± 0.27^a^	2.45 ± 0.23^a^
2	2.5 ± 0.31^ab^	2.175 ± 0.17^ab^	2.25 ± 0.20^b^	2.2 ± 0.18^c^	2.15 ± 0.23^bc^	2.2 ± 0.18^c^	2.375 ± 0.17^a^	2.225 ± 0.52^a^	2.4 ± 0.18^a^
4	2.625 ± 0.17^abc^	2.05 ± 0.20^ab^	2.075 ± 0.22^b^	2.125 ± 0.22^bc^	2.03 ± 0.25^bc^	2.1 ± 0.18^c^	2.22 ± 0.263^a^	2.05 ± 0.20^a^	2.325 ± 0.17^a^
6	2.85 ± 0.23^bc^	1.975 ± 0.27^ab^	1.55 ± 0.20^a^	1.65 ± 0.26^ab^	1.6 ± 0.39^ab^	1.575 ± 0.25^b^	2.175 ± 0.125^a^	1.875 ± 0.22^a^	2.25 ± 0.45^a^
8	3.025 ± 0.17^c^	1.725 ± 0.17^a^	1.35 ± 0.20^a^	1.25 ± 0.23^a^	1.05 ± 0.12^a^	1.1 ± 0.21^a^	2.1 ± 0.185^a^	1.7 ± 0.54^a^	2.175 ± 0.22^a^
*p* values	0.002^***^	0.004^***^	0.000^***^	0.000^***^	0.000^***^	0.000^***^	0.356	0.092	0.628

*Note*: The water tanks with Climbing perch (*Anabas testudineus*) were treated with different water additives and observed for a period of 8 h. Values are presented as mean ± standard deviation (SD). Different superscript letters (e.g., a, b, c) in the same column refers significantly difference between them at 1% level (*p* < 0.01), as determined by one‐way ANOVA.

Abbreviations: ES, electromin saline; MB, methylene blue.

*** refers statistically significant at 1% level.

Similar trends were observed in case of on field experiment; bacterial counts of the treated tanks were found to decrease with time, except in the control tank (initial count, 28 ± 0.216 × 10^4^ CFU/mL, increased to 3.05 ± 0.129 × 10^4^ CFU/mL at the end, after 8 h. The bacterial counts in 1.0 ppm MB treatment were found to decrease significantly (*p *= 0.001) from 2.20 ± 0.221 × 10^4^ to 1.43 ± 0.221 × 10^4^ CFU/mL. Moreover, bacterial counts at 2.0 ppt salt treatment showed to decrease from 2.30 ± 0.216 × 10^4^ to 2.08 ± 0.189 × 10^4^ CFU/mL, and in 2.0 ppt ES, decreased significantly (*p *= 0.005) from 2.25 ± 0.262 × 10^4^ to 1.55 ± 0.341 × 10^4^ CFU/mL. Similar trends were observed in the tanks treated with 2.0 ppt salt + 1.0 ppm MB and 2.0 ppt ES + 1.0 ppm MB, and bacterial counts decreased to 1.93 ± 0.309 × 10^4^ and 1.00 ± 0.210 × 10^4^ CFU/mL, respectively, after 8 h of observation (Table 2).

**TABLE 2:** Changes in viable bacterial counts (CFU/mL) of water in case of on‐field experiment in a supply channel.

	Viable count of bacteria (mean ± SD) × 10^4^ CFU/mL					
		Salt treated	Saline treated	Methylene blue treated	Combination of salt/saline and methylene blue	
Experimental periods (h)	Control	2.0 ppt salt	2.0 ppt ES	1.0 ppm MB	2.0 ppt salt + 1.0 ppm MB	2.0 ppt ES + 1.0 ppm MB
0	2.275 ± 0.22^a^	2.3 ± 0.21^a^	2.325 ± 0.236^b^	2.2 ± 0.21^c^	2.25 ± 0.12^a^	2.325 ± 0.33^c^
2	2.30 ± 0.21^ab^	2.275 ± 0.17^a^	2.25 ± 0.288^b^	2.225 ± 0.22^c^	2.225 ± 0.170^a^	2.1 ± 0.18^c^
4	2.35 ± 0.25^ab^	2.225 ± 0.25^a^	2.125 ± 0.221^ab^	2.1 ± 0.21^bc^	2.2 ± 0.216^a^	1.825 ± 0.22^bc^
6	2.725 ± 0.17^bc^	2.125 ± 0.22^a^	1.8 ± 0.216^ab^	1.625 ± 0.33^ab^	2.025 ± 0.17^a^	1.425 ± 0.27^ab^
8	3.05 ± 0.12^c^	2.075 ± 0.18^a^	1.55 ± 0.341^a^	1.425 ± 0.22^a^	1.925 ± 0.22^a^	1 ± 0.21^a^
*p* values	0.000^***^	0.521	0.005^***^	0.001^***^	0.169	0.000^***^

*Note*: The water tanks with Climbing perch (*Anabas testudineus*) were treated with different water additives and observed for a period of 8 h. Values are presented as mean ± standard deviation (SD). Different superscript letters (e.g., a, b, c) in the same column refers significantly difference between them at 1% level (*p* < 0.01), as determined by one‐way ANOVA.

Abbreviations: ES, electromin saline; MB, methylene blue.

*** refers statistically significant at 1% level.

### 3.3 Changes in Blood Glucose Levels of Fish in the Tanks Treated With Different Chemical Additives

Initial (at 0 h) glucose levels were lower in all the treatments and also similar. Glucose levels were increased with time interval after 8 h, and values were relatively higher in the control tanks of both on‐farm and field experiments, indicating fishes experienced more adverse situations in the control tanks (Table 3). Initially, the mean blood glucose levels were 2.36 ± 0.15 and 5.97 ± 0.4 mmol/L for the fishes of the on‐farm and field experiment, respectively. In the control tanks after 8 h, the blood glucose level was increased to 8.3 ± 0.3 mmol/L for both cases (on‐farm and field). Although the blood glucose levels of the fishes were found to increase in all treatments, the levels were significantly lower in the fishes treated with 1.0 ppm MB and 2.0 ppt ES in case of on‐farm experiment (Table 3; *p *= 0.000). Similarly, in the case of field experiment, blood glucose levels of the fishes were increased in all the treatments after 8 h of transportation. The blood glucose levels were least fluctuated in the fishes treated with 2.0 ppt ES + 1.0 ppm MB (6.7 ± 0.29 mmol/L) followed by 1.0 ppm MB (7.125 ± 0.15 mmol/L) and 2 ppt ES (7.475 ± 0.30 mmol/L) treated tanks (Table 3).

**TABLE 3:** Changes of blood glucose levels (mmol/L) in Climbing perch (*Anabas testudineus*) in case of on‐farm and on field experiments.

Treatment		Blood glucose levels (mmol/L) on‐farm experiment (mean ± SD)	Blood glucose levels (mmol/L) on‐field experiment (mean ± SD)
Average initial level at 0 h		2.36 ± 0.15^a^	5.97 ± 0.4^a^
Values after 8 h of observation	Control	8.3 ± 0.3^e^	8.325 ± 0.34^d^
	2.0 ppt salt	6.4233 ± 0.06^b^	8.175 ± 0.377^cd^
	2.0 ppt ES	6.15 ± 0.14^b^	7.475 ± 0.30^b^
	0.5 ppm MB	6.6633 ± 0.09^b^	Not applicable
	1.0 ppm MB	6.0967 ± 0.18^b^	7.125 ± 0.15^ab^
	1.5 ppm MB	6.9433 ± 0.42^bc^	Not applicable
	2.0 ppt salt + 0.5 ppm MB	7.6333 ± 0.15^cd^	Not applicable
	2.0 ppt salt + 1.0 ppm MB	6.2667 ± 0.035^b^	7.575 ± 0.22^bc^
	2.0 ppt salt + 1.5 ppm MB	7.3667 ± 0.11^d^	Not applicable
	2.0 ppt saline + 1.0 ppm MB	Not applicable	6.7 ± 0.29^a^
	*p* values	0.000^***^	0.000***

*Note*: The test was conducted just before loading (at 0 h) and at the end of the experiment period (after 8 h of observation). Values are presented as mean ± standard deviation (SD). Different superscript letters (e.g., a, b, c) in the same column refers significantly difference between them at 1% level (*p* < 0.01), as determined by one‐way ANOVA.

Abbreviations: ES, electromin saline; MB, methylene blue.

*** refers statistically significant at 1% level.

## 4 Discussion

Live fish transportation is a common practice due to consumers’ preferences and the potential for higher market prices. When a large density of live fish is transported, the water quality deteriorates rapidly, leading to increased stress, accumulation of metabolic wastes, bacterial regrowth and a decline in fish quality as well as market price. This study investigates the effects of various water additives on bacterial growth, water quality parameters and stress reduction in Climbing perch during live transportation.

Water temperature depends on atmospheric temperature. During live transportation of fish, water temperature can be increased (Lima and de Oliviera 2018) or decreased (Gomes et al. 2003). But in this study, it was found that water temperature was slightly fluctuated from 27.5°C to 29°C in all treatments of both laboratory (Figure 2A) and field experiment (Figure 2B), which is optimal for fish and bacterial population (Bhuiyan et al. 2022). Initial pH values of the tanks were ∼8.0, which was found to decrease slightly with time for both the laboratory (Figure 3A) and field experiments (Figure 3B); however, within the optimum range (Ahmed et al. 2015), the pH values in the control tank dropped quickly as transportation time increased, which may be due to the accumulation of CO_2_ in the fish tanks, which may eventually generate carbonic acid (Hong et al. 2019; Wurts 2003; Singh et al. 2004). However, in the treated tanks, the pH levels remained stable in both the cases of on‐farm (Figure 3A) and field (Figure 3B) experiments.

Dissolved oxygen is a limiting factor in any fish‐holding system (Couderc et al. 2015) and decreases from time to time (Cooke et al. 2004). High metabolic activity and decomposition can lower DO levels in the water. To reduce stress during live fish transportation, it is important to maintain enough DO throughout the trip. In this study, tanks treated with chemical additives (especially with 2.0 ppt ES, 1.0 ppm MB and 2.0 ppt salt + 1.0 ppm MB) showed less fluctuation in DO concentration in both on‐farm (Figure 4A) and field (Figure 4B) experiments. Moreover, in the on‐field trial, the lowest depletion (4 ppm DO after 8 h) of DO was found in the water treated with 2.0 ppt ES + 1.0 ppm MB, indicating that this dose is effective to keep the DO concentration suitable (Figure 4B). More or less similar results were also reported by Faruk et al. (2019), Waichman et al. (2001) and Grottum et al. (1997).

Ammonia (NH_3_) builds up due to protein metabolism of the fish in transport water and bacterial decomposition of organic matter (Roberts and Shepherd 1997; Randall and Tsui 2002). The concentrations of ammonia increased gradually in all the tanks during farm and field experiments. Ammonia production was comparatively higher in the untreated water, whereas it was lower in the treated tanks (Figure 5A,B). However, relatively less concentration of ammonia was observed in the water treated with 2.0 ppt ES + 1.0 ppm MB in field experiment (Figure 5B). Thus, it is clear that use of chemical additives reduces ammonia concentrations in water used during live transportation of Climbing perch (Figure 5A,B).

Viable cell count was found to increase especially after 8 h during on‐farm experiment in control tanks (Table 1). A similar trend was also observed during on‐field experiment (Table 2). During live transportation, metabolisms and movements may be resulted bacterial regrowth (Hossain et al. 2021). Poor water quality causes burn of the slime coat or stresses the Climbing perch making it more susceptible to the diseases caused by bacteria (Dobšíková et al. 2006; Arshad et al. 2006; Al‐Niaeem et al. 2009; Bolivar et al. 2004; Faruk et al. 2019). In case of on‐farm experiment, all the tanks treated with 2.0 ppt salt, 2.0 ppt ES and 1.0 ppm MB showed significantly lower bacterial counts after 8 h observation (Table 1). Among the three combined treatments, 2.0 ppt salt + 1.0 ppm MB showed relatively better results in reducing bacterial loads (Table 1). In case of on‐field trial, tanks treated with 1.0 ppm MB and 2.0 ppt ES showed more effectiveness in lowering bacterial counts; however, tank treated with 2.0 ppt ES + 1.0 ppm MB showed significantly lower bacterial counts (Table 2).

MB is a redox dye which raises the oxygen consumption of cells. Here, MB acts as an inhibitor of bacteria and fungi (Schaperclaus 1992). Again, salt is inexpensive, readily available, and when properly administered, safe for use which has antimicrobial properties (Kitancharoen et al. 1997). Many studies have documented the advantages of using salt during and after the transport of various species (Tomasso et al. 1980; Johnson and Metcalf 1982; Luz and Favero 2024; Mazik et al. 1991; Barton and Zitzow 1995; Cech et al. 1996; Swanson et al. 1996). Commercially available ES claims that it can maintain the fluid and electrolyte balance, acid–base balance in the body. It fulfils the deficiency of electrolyte and minerals. It prevents stress (fluid loss) during transportation. It can be the reason why commercially available saline can suppress bacterial load more than table salt (NaCl). Both MB and saline were found effective individually but the combined use of saline and MB showed best result in terms of lowering final bacterial load (Faruk et al. 2019).

Transported fish are often exposed to multiple stressors within a short duration. Stress can also play a major role in the susceptibility of fish to disease (Winton 2001). Measuring the circulating level of cortisol and/or blood glucose level is used as an indicator to evaluate the degree of stress experienced by fish (Barton 2002; Pankhurst 2011; Pottinger 2008). In this study, blood glucose levels were measured to assess fish stress during live transportation with and without water additives. Both on‐farm and field experiments showed that the initial average glucose levels were lower, gradually increasing with prolonged transport. However, glucose levels were significantly higher in fish from the untreated control tanks compared to those in tanks with water additives. Here too, the combination of salt or saline with MB showed better results (Table 3). The findings indicate that using water additives such as salt, ES and MB during the live transportation of Climbing perch positively impacts water quality, reduces bacterial regrowth and lowers physiological stress. Additionally, combining MB with salt/ES proved even more effective and could be adopted by fish farmers and traders to minimize fish quality deterioration, mortality and financial losses.

## 5 Conclusion

The results rebuilt that using water additives such as salt, ES and MB during the live transportation of Climbing perch enhanced the transport water quality, reduced bacterial regrowth and lowered physiological stress of the fishes. Additionally, combined use of MB with ES provided even better results and could be adopted by fish farmers and traders to minimize fish quality deterioration, mortality and financial losses.

## Author Contributions

Md. Nurul Haider and Md. Mubrack Hossain formulated the research idea and acquired the funds. Md. Nurul Haider, Md. Mubrack Hossain and Md. Naim Uddin designed the experiments. Maliha Afsana and Md. Nazmul Islam Rifat carried it out and conducted the laboratory analysis. Maliha Afsana prepared the first draft of this manuscript. Md. Nurul Haider guided all the activities and critically revised and submitted this manuscript.

## Ethics Statement

The study protocol was approved, and the research was performed according to the guidelines of the Animal Welfare and Experimentation Ethics Committee (AWEEC) of Bangladesh Agriculture University in Mymensingh [AWEEC/BAU/2024 (Hong et al. 2019)].

## Conflicts of Interest

The authors declare no conflicts of interest.

## Peer Review

The peer review history for this article is available at https://www.webofscience.com/api/gateway/wos/peer‐review/10.1002/vms3.70544.

## Data Availability

Data will be made available on request.
